# Prevalence of Sarcopenia and Its Defining Components in Non-alcoholic Fatty Liver Disease Varies According to the Method of Assessment and Adjustment: Findings from the UK Biobank

**DOI:** 10.1007/s00223-024-01212-5

**Published:** 2024-04-28

**Authors:** Christine L. Freer, Elena S. George, Sze-Yen Tan, Gavin Abbott, David Scott, Robin M. Daly

**Affiliations:** 1 Institute for Physical Activity and Nutrition, School of Exercise and Nutrition Sciences, Deakin University, Geelong, VIC Australia; 2 School of Clinical Sciences at Monash Health, Monash University, Clayton, Australia

**Keywords:** Sarcopenia, Muscle mass, Muscle strength, Physical function, Non-alcoholic fatty liver disease, UK Biobank

## Abstract

Sarcopenia may increase non-alcoholic fatty liver disease (NAFLD) risk, but prevalence likely varies with different diagnostic criteria. This study examined the prevalence of sarcopenia and its defining components in adults with and without NAFLD and whether it varied by the method of muscle mass assessment [bioelectrical impedance (BIA) versus dual-energy X-ray absorptiometry (DXA)] and adjustment (height^2^ versus BMI). Adults (*n* = 7266) in the UK Biobank study (45–79 years) with and without NAFLD diagnosed by MRI, were included. Sarcopenia was defined by the 2018 European Working Group on Sarcopenia in Older People definition, with low appendicular skeletal muscle mass (ASM) assessed by BIA and DXA and adjusted for height^2^ or BMI. Overall, 21% of participants had NAFLD and the sex-specific prevalence of low muscle strength (3.6–7.2%) and sarcopenia (0.1–1.4%) did not differ by NAFLD status. However, NAFLD was associated with 74% (males) and 370% (females) higher prevalence of low ASM when adjusted for BMI but an 82% (males) to 89% (females) lower prevalence when adjusted for height^2^ (all *P* < 0.05). The prevalence of impaired physical function was 40% (males, *P* = 0.08) to 123% (females, *P* < 0.001) higher in NAFLD. In middle-aged and older adults, NAFLD was not associated with a higher prevalence of low muscle strength or sarcopenia but was associated with an increased risk of impaired physical function and low muscle mass when adjusted for BMI. These findings support the use of adiposity-based adjustments when assessing low muscle mass and the assessment of physical function in NAFLD.

**Supplementary Information:**

The online version contains supplementary material available at 10.1007/s00223-024-01212-5.

## Introduction

Non-alcoholic fatty liver disease (NAFLD) is now estimated to affect approximately 30% of the adult population [1] with the growing burden of NAFLD strongly related to modifiable lifestyle factors such as poor dietary habits and physical inactivity [2]. Low skeletal muscle mass, a key component of the disease sarcopenia (defined as low muscle mass, strength and function) has emerged as an independent risk factor for NAFLD and its severity, with some evidence that low muscle mass is associated with a 30% increased risk of NAFLD [3]. While muscle loss and NAFLD share several common underlying risk factors including systemic inflammation and insulin resistance in their pathophysiology and progression [4], there are mixed findings from the limited studies available with regard to the prevalence of low muscle mass in adults with NAFLD.

The prevalence of low muscle mass in NAFLD adults has reported to range from 0 to 63% [5–9]. This heterogeneity is likely due to differences in participant demographics, the method of assessment for muscle mass [dual-energy X-ray absorptiometry (DXA) or bioelectrical impedance analysis (BIA)], the method of muscle mass adjustment to account for differences in body size (e.g. height^2^, BMI or weight) and/or the cut-off values used to define low muscle mass [10, 11]. For example, a study in 156 older adults with NAFLD, in which 72% were obese, reported a significantly higher prevalence of low appendicular skeletal muscle mass (ASM) when adjusted for BMI compared to height^2^ (26% vs 13.5%) [6]. This is likely explained by previous research showing that adiposity can mask the prevalence of low muscle mass as higher BMI has been associated with greater absolute but lower relative muscle mass [12, 13]. Thus, in obesity-related metabolic disorders such as NAFLD it may be more relevant to use adiposity-adjusted definitions and cut-offs for defining low muscle mass and the prevalence of sarcopenia.

Current operational definitions of sarcopenia have evolved to incorporate low muscle mass in combination with low muscle strength and impaired physical function, with the 2018 European Working Group of Sarcopenia in Older People (EWGSOP2) definition being the most widely utilised [10]. Of the available studies assessing two or more components of sarcopenia in adults with NAFLD, the prevalence has ranged from 0.5 to 38.6% but these studies have used different definitions, cut-off points, and adjustment methods which makes comparison challenging [12, 14, 15]. To our knowledge, only two studies have assessed the prevalence of sarcopenia in those with NAFLD (age 26–73 years) using EWGSOP2, reporting a prevalence ranging from 0.0 to 1.6% [9, 16]. Since NAFLD has a higher likelihood of obesity [2], low muscle mass and sarcopenia prevalence may be underestimated given the EWGSOP2 definition recommends adjustment of muscle mass for height^2^. Therefore, the primary aim of this study was to estimate the prevalence of sarcopenia and its defining components (low muscle strength, low muscle mass or impaired physical function) in middle-aged and older adults with NAFLD compared to those without NAFLD. Secondary aims were to determine whether the prevalence differs according to methods of muscle mass adjustment (height^2^ versus BMI) and methods of assessment (BIA versus DXA), and to evaluate the level of agreement between these adjustment methods and assessments.

## Methods

### Study Design

This study was a secondary analysis of cross-sectional data from the UK Biobank resource under application number 73818. The UK Biobank is a long-term prospective study following the health of over 500,000 adults living in the UK aged between 40 and 69 years, first recruited between 2006 and 2010 [17]. Participants were recruited via centrally coordinated identification and invitation from population-based registers (including the National Health Service). At baseline, health-related information (socio-demographics, lifestyle, family history, health, and medical history) was obtained through self-completed touch screen questionnaires. Anthropometry, body composition (assessed by BIA) and grip strength were also assessed, and biological samples taken [17]. In 2014 the UK Biobank imaging enhancement protocol was initiated incorporating abdominal MRI and full body DXA scans [17], with data from the first 10,012 participants who completed the imaging component included in this analysis. The UK Biobank study was approved by the Northwest Multicentre Research Ethics Committee and performed in accordance with the ethical standards laid down in the 1964 Declaration of Helsinki and its later amendments. Informed consent was given by all participants at the time of recruitment.

### Participants

**Fig. 1:**
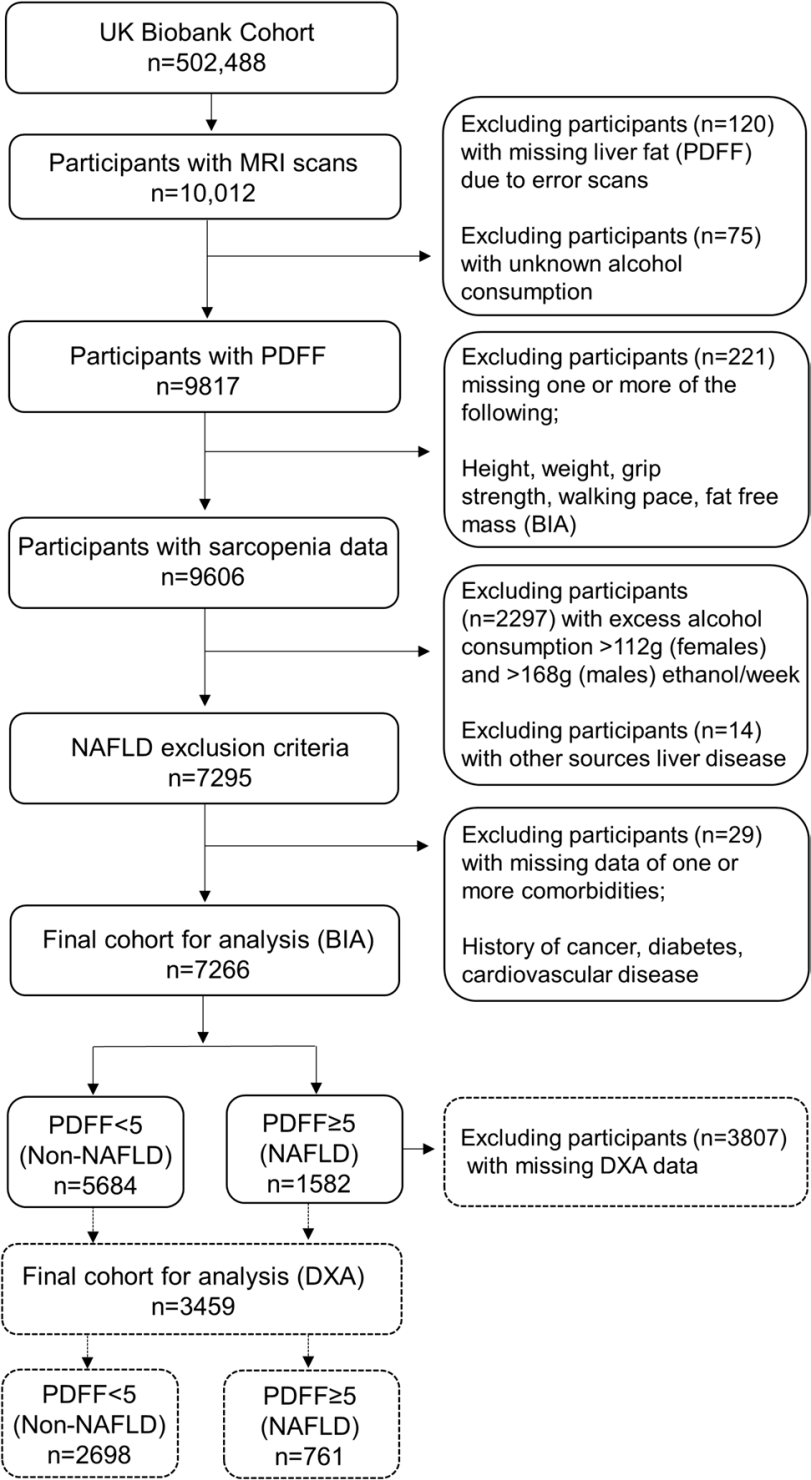
Overview of study cohort selection from UK Biobank. BIA, bioelectrical impedance analysis; DXA, dual-energy X-ray absorptiometry; NAFLD, non-alcoholic fatty liver disease; PDFF, proton density fat fraction

From a total of 502,488 participants enrolled in the UK Biobank study, 10,012 had available MRI liver imaging scans to quantify NAFLD. Of these, participants were excluded if they had missing MRI derived liver proton density fat fraction (PDFF) (*n* = 120) and/or unknown alcohol consumption (*n* = 75). Participants with excessive weekly alcohol consumption (*n* = 2297) (women > 112 g ethanol; men > 168 g ethanol), presence of other causes of liver disease (hepatitis B, hepatitis C, alcoholic liver disease or toxic liver diseases, *n* = 14), or missing data on the presence of one or more comorbidities (cancer, type 2 diabetes, cardiovascular disease, *n* = 29), were excluded. For investigation of sarcopenia, 221 participants were excluded due to missing data for one or more of the following variables: height, weight, grip strength, walking pace or fat free mass (FFM) assessed via BIA (Fig. 1). A subset of 3459 participants had a whole body DXA scan, and their demographic and health/medical characteristics were similar to the full cohort (*n* = 7266) that had a BIA assessment (Supplementary Table 1). For regression analysis, participants were further excluded if there were missing data for smoking status (*n* = 14) and/or physical activity (*n* = 1092), which were used as covariates.

### Measures

#### Demographics and Health/Medical History

Information on demographic characteristics [age, sex, and ethnicity (white: British, White and Black Caribbean, Indian, Caribbean) or (non-white: mixed, Asian or Asian British, Black or Black British, Chinese, other ethnic group)] and history of diseases/illnesses (comorbidities) were collected via a touchscreen questionnaire. For the latter, participants were asked if they had ever been diagnosed by a doctor with the following: diabetes, vascular/heart problems (heart attack, angina, stroke, high blood pressure) or cancer. Information about smoking status (never, previous, current) and alcohol intake (frequency and average weekly intake of red wine, white wine, beer, cider, spirits, fortified wine, and other drinks) was also derived via the touchscreen questionnaire. The NHS alcohol unit’s definition was used to calculate the weekly number of alcohol units and intake (in grams) to assess excessive alcohol consumption [18].

#### Liver Imaging and NAFLD Diagnosis

Liver images via MRI were acquired at the UK Biobank Imaging Centre. Hepatic fat was quantified via MRI (Siemens 1.5T MAGNETOM Area) derived PDFF using a 6-min dual-echo Dixon vibe protocol [19]. As reported previously [20], a single transverse slice was captured, taken at the porta hepatitis. Liver images were analysed using Liver*MultiScan*™ Discover software (Perspectum Diagnostics) to obtain measures of liver fat % and MR echoes used to create PDFF maps using a three-point DIXON technique [21]. NAFLD was defined as > 5% liver fat in the absence of excessive alcohol consumption and presence of other causes of liver disease. MRI derived PDFF has been shown to correlate strongly with histopathological liver triglyceride content [22].

#### Definition of Sarcopenia

Sarcopenia was defined as probable sarcopenia [low muscle (grip) strength] and confirmed with the addition of low ASM for BIA and low appendicular lean mass (ALM) for DXA, with severe sarcopenia identified when impaired physical function was also present. The diagnostic criteria and cut-off points recommended by the EWGSOP2 guidelines were used for low grip strength and low ASM/ALM [10] as indicated below.

#### Muscle (Lean) Mass

Low ASM was estimated from FFM (sum of arms and legs) assessed by BIA using a Tanita BC418MA body composition analyser. Since BIA does not measure ASM, it was estimated using the following equation developed by Dodds et al. [23] using data from UK Biobank participants who had a total body DXA scan to evaluate appendicular lean mass: ASM (kg) = (0.958 × appendicular FFM (kg) − (0.166 × S) −0.308, with S being given the value 0 if female or 1 if male. In addition, a subset of UK Biobank participants had a total body DXA scan (GE-Lunar, Madison W1) performed by a trained radiographer which was used to assess total body and regional (arms, legs, and trunk) lean mass. Lean mass for the arms and legs was summed to calculate ALM (kg).

Two techniques were used to adjust ASM and ALM for body size: (1) ASM/ALM adjusted for height (in metres squared); and (2) ASM/ALM adjusted for BMI. The following sex-specific cut-points were used for both the BIA and DXA measures to indicate low muscle mass: (1) Low ASM or ALM divided by height^2^; < 7.0 kg/m^2^ for males; < 5.5 kg/m^2^ for females; [10] and (2) low ASM or ALM divided by BMI; < 0.789 for males; < 0.512 for females [11].

#### Muscle (Grip) Strength

Low muscle strength was assessed via hand grip strength using a Jamar J00105 hydraulic hand dynamometer (Lafayette Instrument, USA). Participants were instructed to sit upright in a chair with elbows at 90° flexion with forearms placed on arm rests in the mid prone position. Hand grip strength was measured once for both the left and right arm using a 3-s maximum grip effort. The maximum value (in kg) for either arm was used as the result. Low muscle strength was defined as < 27 kg for males and < 16 kg for females [10].

#### Physical Function

Impaired physical function for the assessment of sarcopenia is typically based on an assessment of gait speed [10], but since the UK Biobank did not include a physical assessment of function, self-reported usual walking pace (1 unable to walk, 2 slow, 3 steady, or 4 brisk) was assessed via the touchscreen questionnaire and used as a surrogate estimate of physical function. As reported in previous studies using the UK Biobank data [23], impaired physical function was defined as unable to walk or walked at a slow pace.

#### Physical Activity

Physical activity was assessed via questions adapted from the validated short International Physical Activity Questionnaire (IPAQ) [24]. Questions encompassed the frequency, intensity and duration of walking and moderate and vigorous physical activity. Total physical activity expressed as MET min/week was determined by calculating the energy expended for the following activities: time spent in vigorous and moderate activity and walking. MET min/week = Walk (METs*min*days) + Moderate activity (METs*min*days) + Vigorous (METs*min*days [24].

#### Anthropometry

Height (in cm) was measured using a Seca stadiometer (Seca, Hamburg, Germany). Weight (in kg) was assessed using a Tanita BC418MA body composition analyser. Body mass index (BMI; kg/m^2^) was assessed from weight (kg) divided by height (m^2^).

### Statistical Analysis

All analyses were performed using Stata Statistical software version 17.0. Descriptive statistics are presented as mean and SD or frequency counts and proportions (percentages). Independent t-tests (continuous variables) and chi-square tests (categorical variables) were used to compare baseline characteristics and the prevalence of low muscle strength, low ASM/ALM, and impaired physical function between participants with and without NAFLD. All results are presented for males and females separately. Generalised linear models, using a binomial family and log link function were used to estimate prevalence ratios with 95% confidence intervals for predicting low muscle strength (probable sarcopenia), low ASM/ALM, and impaired physical function, dependent upon the presence of NAFLD. Models were analysed unadjusted (model 1) and adjusted for age, physical activity, smoking status and number of comorbidities (model 2) and additionally adjusted for BMI (model 3). For low ASM/ALM outcomes, model 3 was not used, as ASM/ALM was already adjusted for height^2^ or BMI. Due to very small numbers of participants with confirmed and severe sarcopenia, no model was fitted for this exposure variable. Separate models were fitted for each individual exposure variable. The models included the exposure variable sex and their interaction, to estimate prevalence ratios for males and females separately as well as estimate sex differences in prevalence ratios. The Kappa test was used to evaluate the level of agreement between low ALM/ASM adjusting for height^2^ and BMI and sarcopenia using DXA and BIA. The strength of agreement (Kappa coefficients) was interpreted as < 0 no, 0–0.20 slight, 0.21–0.39 fair, 0.40–0.59 moderate, 0.61–0.80 substantial, and ≥ 0.81 almost perfect agreement [25]. Positive and negative predictive agreements were used to reflect the proportion of participants who were classified into low muscle mass or sarcopenia criteria (PPA%) or not having low muscle mass or sarcopenia (NPA%) [26]. Statistical significance was set at *P* < 0.05.

## Results

### Participant Characteristics

The characteristics of the participants by NAFLD status and sex are shown in Table 1. The mean ± SD age of participants was 62.7 ± 7.6 years with 54.9% female. Overall, 21.8% of the participants (*n* = 1582) had NAFLD, with a higher proportion in males compared to females (27.0% vs 17.5%). The mean PDFF for females and males with NAFLD was 11.3 ± 6.3% and 10.5 ± 10.1%, respectively. Participants with NAFLD were more likely to be obese (males 49% vs 13%; females 43% vs 10%, respectively) and have lower physical activity levels (all *P* < 0.001) and a higher prevalence of comorbidities.

**Table 1:** Characteristics of the study population according to NAFLD status and sex

	Females		*P* value	Males		*P* value
	Non-NAFLD	NAFLD		Non-NAFLD	NAFLD	
*n*	3294	697		2390	885	
Age (years)	61.9 ± 7.4	62.9 ± 7.0	< 0.001	63.7 ± 7.8	63.0 ± 7.7	0.020
Height (cm)	162.9 ± 6.2	161.9 ± 6.2	< 0.001	175.8 ± 6.6	175.8 ± 6.5	0.933
Weight, kg	67.2 ± 11.6	80.9 ± 14.7	< 0.001	80.2 ± 11.6	92.8 ± 14.8	< 0.001
BMI, kg/m^2^	25.3 ± 4.2	30.8 ± 5.1	< 0.001	25.9 ± 3.3	30.0 ± 4.2	< 0.001
Ethnicity^a^, *n* (%)						
White	3065 (93.2)	656 (94.4)	0.570	2234 (93.6)	832 (94.5)	0.295
Non-White	223 (6.8)	39 (5.6)		153 (6.4)	49 (5.5)	
Smoking status, *n*						
Never, *n* (%)	2310 (70.2)	461 (66.3)	0.126	1534 (64.3)	521 (59.1)	0.012
Previous, *n* (%)	885 (26.9)	211 (30.4)		771 (32.3)	317 (36.0)	
Current, *n* (%)	94 (2.9)	23 (3.3)		82 (3.4)	43 (4.9)	
Comorbidities^b^, *n* (%)						
1	826 (25.1)	263 (37.7)	< 0.001	772 (32.3)	340 (38.4)	< 0.001
≥ 2	99 (3.0)	81 (11.6)		156 (6.5)	125 (14.1)	
PA, MET- min/week^c^	2961 ± 3249	2375 ± 2477	< 0.001	3248 ± 3722	2330 ± 2966	< 0.001
BIA						
ASM, kg	17.5 ± 2.1	19.2 ± 2.7	< 0.001	25.0 ± 3.3	27.8 ± 4.2	< 0.001
ASM/Ht^2^	6.60 ± 0.70	7.33 ± 0.35	< 0.001	8.08 ± 0.88	8.98 ± 1.13	< 0.001
ASM/BMI	0.700 ± 0.080	0.629 ± 0.062	< 0.001	0.970 ± 0.098	0.931 ± 0.091	< 0.001
DXA^d^						
ALM, kg	17.2 ± 2.5	18.5 ± 3.0	< 0.001	25.0 ± 3.3	27.1 ± 4.1	< 0.001
ALM/Ht^2^	6.47 ± 0.79	7.10 ± 1.00	< 0.001	8.06 ± 0.87	8.72 ± 1.04	< 0.001
ALM/BMI	0.683 ± 0.096	0.608 ± 0.078	< 0.001	0.966 ± 0.117	0.908 ± 0.099	< 0.001
Grip strength, kg	24.6 ± 5.8	24.1 ± 6.1	0.062	40.1 ± 8.3	40.2 ± 8.2	0.685
Physical function, *n* (%)						
Slow	117 (3.6)	75 (10.8)	< 0.001	77 (3.2)	50 (5.6)	< 0.001
Steady	1596 (48.4)	461 (66.2)		1129 (47.2)	524 (59.2)	
Brisk	1580 (48.0)	160 (23.0)		1184 (49.5)	311 (35.1)	

Values represent mean ± SD or frequency counts and proportions (%) unless otherwise indicated

*ALM* appendicular lean mass, *ASM* appendicular skeletal muscle mass, *BIA* bioelectrical impedance analysis, *BMI* body mass index, *DXA* dual-energy X-ray absorptiometry, *H*^*2*^ height in metres squared, *MET* metabolic equivalents, *NAFLD* non-alcoholic fatty liver disease, *PA* physical activity, *PDFF* proton density fat fraction, *P* *P* value

^a^Number of participants with ethnicity data (*n* = 7251)

^b^Comorbidities included diagnosed diabetes, vascular/heart problems (heart attack, angina, stroke, high blood pressure) and cancer

^c^Number of participants with PA data (*n* = 6169)

^d^Number of participants with DXA data (*n* = 3459)

Regarding the components of sarcopenia, the mean absolute ASM/ALM (by BIA and DXA) was 1.3–2.8 kg higher (all, *P* < 0.001) in both males and females with NAFLD. Similarly, mean ASM/ALM adjusted for height was higher (8.1–11.1%, all, *P* < 0.001) in males and females with NAFLD. In contrast, ASM/ALM adjusted for BMI was 4.1–11.1% lower (all, *P* < 0.001) in both males and females with NAFLD. While there was a trend (*P* = 0.062) for grip strength to be 0.5 kg lower in females with NAFLD, a significantly higher proportion of males and females with NAFLD reported a slow or steady compared to brisk walking pace indicative of impaired physical function (both *P* < 0.001; Table 1).

### Prevalence of Sarcopenia and Its Components

The sex-specific estimated prevalence of low muscle strength, mass (ASM/ALM) and impaired physical function in those with and without NAFLD and their prevalence ratios according to NAFLD are reported in Table 2. Similar results were observed in the subset of participants with both DXA and BIA scans (Supplementary Table 2).

**Table 2:** Sex-specific prevalence of low muscle strength, low appendicular skeletal muscle mass according to BIA and DXA adjusting for height and BMI, and impaired physical function, in those with and without NAFLD and prevalence ratios (95% CI) according to NAFLD

	Non-NAFLD	NAFLD	Prevalence ratio (95% CI)					
			Model 1	*P* value	Model 2	*P* value	Model 3	*P* value
Females	*n* = 3294	*n* = 697						
Low muscle strength, *n* (%)	158 (4.8)	50 (7.2)	1.41 (0.96, 2.08)	0.080	1.33 (0.90, 1.96)	0.149	1.46 (0.96, 2.21)	0.079
Low muscle mass, *n* (%)								
* ASM/Ht*^*2 (BIA)*^	78 (2.4)	4 (0.6)	0.08 (0.01, 0.59)	0.013	0.08 (0.01, 0.57)	0.012	*–*	*–*
* ASM/BMI*^*(BIA)*^	12 (0.4)	15 (2.2)	5.10 (1.92, 13.54)	0.001	4.70 (1.79, 12.25)	0.002	*–*	*–*
Low muscle mass^a^, *n* (%)								
* ALM/Ht*^*2 (DXA)*^	126 (8.0)	8 (2.3)	0.19 (0.07, 0.51)	0.001	0.18 (0.07, 0.49)	0.001	*–*	*–*
* ALM/BMI*^*(DXA)*^	41 (2.6)	26 (7.6)	2.55 (1.41, 4.61)	0.002	2.21 (1.22, 4.02)	0.009	*–*	*–*
Impaired function, *n* (%)	117 (3.6)	75 (10.8)	2.65 (1.84, 3.80)	< 0.001	2.23 (1.56, 3.20)	< 0.001	1.02 (0.68, 1.52)	0.923
Males	*n* = 2390	*n* = 885						
Low muscle strength, *n* (%)	109 (4.6)	32 (3.6)	0.73 (0.48, 1.13)	0.161	0.77 (0.50, 1.19)	0.238	0.83 (0.52, 1.32)	0.422
Low muscle mass, *n* (%)								
* ASM/Ht*^*2 (BIA)*^	227 (9.5)	11 (1.2)	0.11 (0.05, 0.22)	< 0.001	0.11 (0.06, 0.23)	< 0.001	*–*	*–*
* ASM/BMI*^*(BIA)*^	59 (2.5)	39 (4.4)	1.65 (1.07, 2.53)	0.023	1.74 (1.12, 2.70)	0.013	*–*	
Low muscle mass^a^, *n* (%)								
* ALM/Ht*^*2 (DXA)*^	112 (10.0)	13 (3.1)	0.29 (0.16, 0.53)	< 0.001	0.29 (0.16, 0.54)	< 0.001	*–*	*–*
* ALM/BMI*^*(DXA)*^	67 (6.0)	50 (12.0)	1.60 (1.08, 2.37)	0.018	1.59 (1.08, 2.35)	0.019	*–*	*–*
Impaired function, *n* (%)	77 (3.2)	50 (5.6)	1.68 (1.14, 2.47)	0.008	1.40 (0.95, 2.07)	0.086	0.77 (0.52, 1.15)	0.205

Values are presented as number and proportions (%) or the prevalence ratio with the 95% confidence intervals (CI)

*ALM* appendicular lean mass, *ASM* appendicular skeletal muscle mass, *BIA* bioelectrical impedance analysis, *BMI* body mass index, *DXA* dual-energy X-ray absorptiometry, *Ht*^*2*^ height in metres squared

^a^Number of participants with DXA data: females *n* = 1579 non-NAFLD and *n* = 344 NAFLD; males *n* = 1119 non-NAFLD and *n* = 417 NAFLD

Model 1: unadjusted. Model 2: adjusted for age, physical activity, presence of comorbidities and smoking status. Model 3: adjusted for age, physical activity, presence of comorbidities, smoking status and BMI

#### Low Muscle Strength (Probable Sarcopenia)

The estimated prevalence of low muscle strength was not statistically different for those with and without NAFLD ranging from 3.6 to 7.2%, and there was no significant sex interaction (*P* = 0.066) for the prevalence ratio (Table 2).

#### Low Muscle Mass

Using BIA, the estimated prevalence of low muscle mass (ASM) adjusting for height^2^ was greater in non-NAFLD than NAFLD females (2.4% vs 0.6%) and males (9.5% vs 1.2%), with an estimated 92–89% (both *P* < 0.05 for model 2) lower prevalence in males and females with NAFLD, respectively. In contrast, the prevalence of low muscle mass (ASM) adjusted for BMI was higher in both females (2.2% vs 0.4%) and males (4.4% vs 2.5%) with NAFLD, with an associated 74% and 370% (both *P* < 0.05 to < 0.01 for model 2) higher prevalence in males and females, respectively, with no significant sex interaction (*P* = 0.068). Using DXA, similar significant findings between NALFD and non-NAFLD males and females were observed for the prevalence of low muscle mass (ALM) after adjusting for height^2^ and BMI. However, the overall prevalence of low muscle mass for both NAFLD and non-NAFLD participants was 1.1 to 6.5-fold higher based on DXA compared to BIA (Table 2).

#### Impaired Physical Function

The estimated prevalence of impaired physical function was 2-3-fold greater in both females and males with NAFLD, with the prevalence ratio (model 2) being 40% (*P* = 0.09) higher in males and 123% (*P* < 0.001) higher in females with NAFLD compared without NAFLD, with no significant sex interaction (*P* = 0.083) (Table 2).

#### Sarcopenia and Severe Sarcopenia

**Fig. 2:**
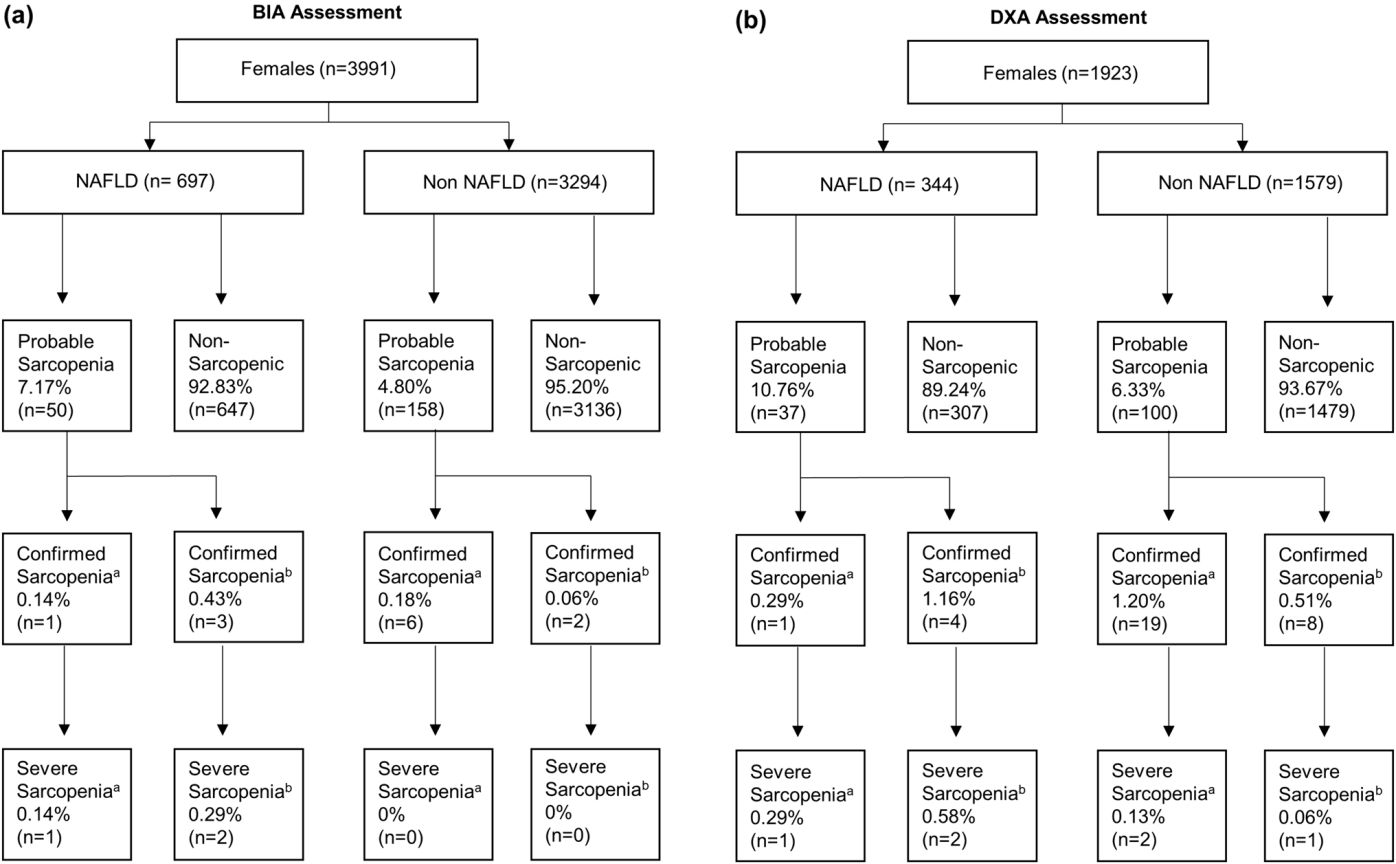
Prevalence of low muscle strength (probable sarcopenia), sarcopenia, and severe sarcopenia in females with and without NAFLD according to BIA (panel a) and DXA (panel b) with figures presenting proportions for each group (NAFLD and non-NAFLD). BIA, bioelectrical impedance analysis; DXA, dual-energy X-ray absorptiometry; NAFLD, non-alcoholic fatty liver disease. ^a^ Low ASM/ALM adjusted for height^2^, ^b^ Low ASM/ALM adjusted for BMI

**Fig. 3:**
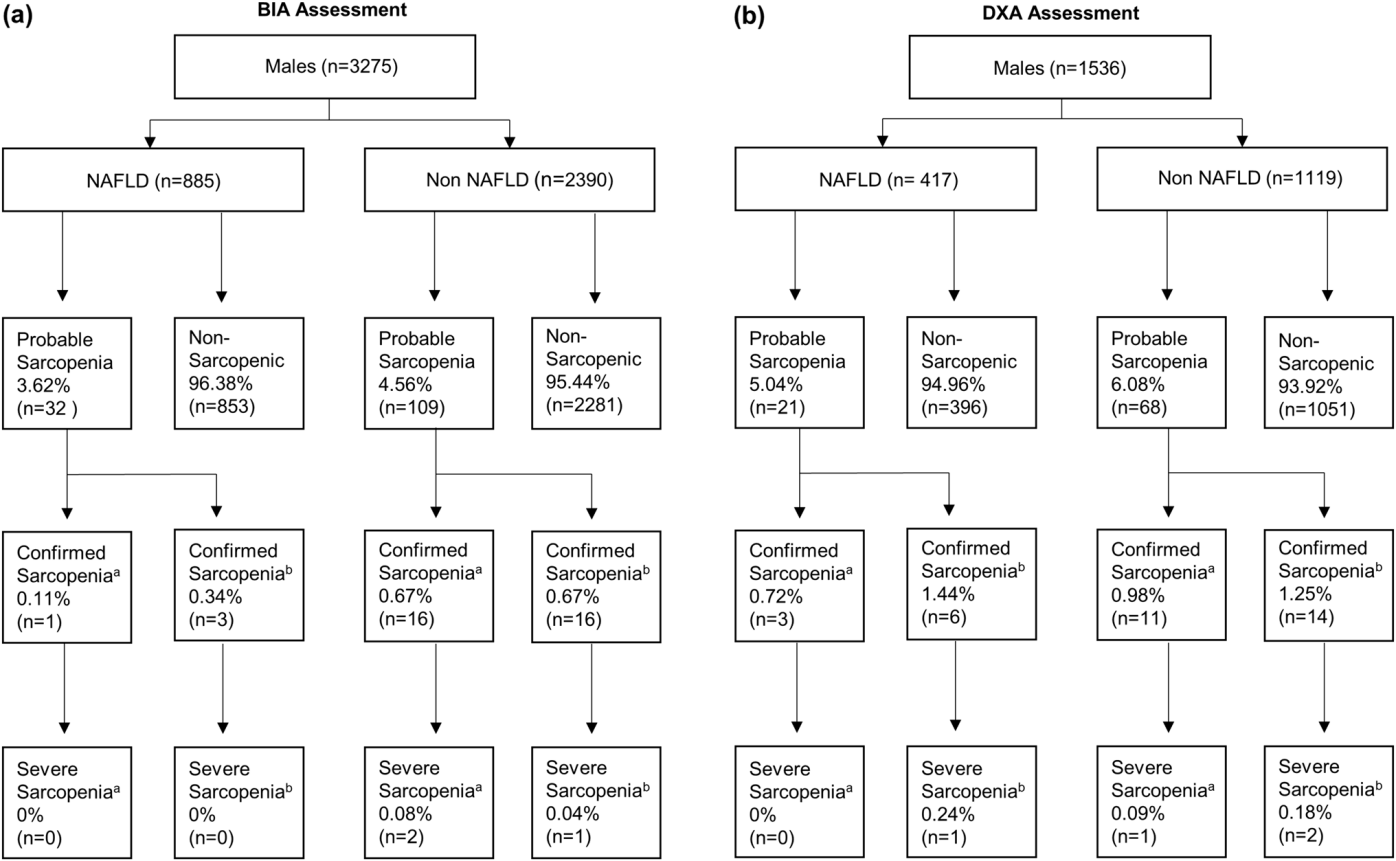
Prevalence of low muscle strength (probable sarcopenia), sarcopenia, and severe sarcopenia in males with and without NAFLD according to BIA (panel a) and DXA (panel b) with figures presenting proportions for each group (NAFLD and non- NAFLD). BIA, bioelectrical impedance analysis; DXA, dual-energy X-ray absorptiometry; NAFLD, non-alcoholic fatty liver disease. ^a^ Low ASM/ALM adjusted for height2, ^b^ Low ASM/ALM adjusted for BMI

Regardless of NAFLD status, the overall prevalence of confirmed sarcopenia was low for both females (0.14–0.43%) (Fig. 2) and males (0.11–0.67%) in this study when ASM was measured by BIA (Fig. 3). Similar findings (albeit slightly higher prevalence’s) were observed when ALM was assessed by DXA (females 0.29–1.20%; males 0.72–1.44%). The overall prevalence of severe sarcopenia (inclusive of impaired function) ranged from 0% to 0.58%, regardless of sex or the method of assessment or adjustment.

### Agreement Between the Body Composition Methods of Assessment and Adjustment

Overall, the level of agreement for low muscle mass and sarcopenia based on the method of adjustment (height^2^ versus BMI) using either BIA or DXA was poor (Kappa, 0.00–0.08; PPA%, 0.0–12.0%) (Table 3). In contrast, agreement for low muscle mass and sarcopenia according to the method of assessment (BIA versus DXA) whether adjusted for height^2^ or BMI was moderate (Kappa, 0.38–0.58). The NPA% was high for all criteria.

**Table 3:** Agreement for the diagnosis of low muscle mass and sarcopenia according to the muscle mass method of adjustment (ASM and ALM divided by height^2^ versus BMI) and by the body composition technique (BIA versus DXA)

	Low muscle mass				Sarcopenia			
	PPA%	NPA%	Kappa ± SE	*P* value	PPA%	NPA%	Kappa ± SE	*P* value
ASM/Ht^2^ vs. BMI^(BIA)^	5.2	96.1	0.01 ± 0.02	0.303	0.0	99.7	0.00 ± 0.02	0.594
ALM/Ht^2^ vs. BMI^(DXA)^	12.0	92.8	0.04 ± 0.02	0.009	9.4	99.1	0.08 ± 0.02	< 0.001
BIA vs. DXA^(Ht2)^	35.5	98.7	0.44 ± 0.02	< 0.001	32.4	100	0.48 ± 0.01	< 0.001
BIA vs. DXA^(BMI)^	26.1	99.7	0.38 ± 0.01	< 0.001	43.8	99.9	0.58 ± 0.02	< 0.001

*ALM* appendicular lean mass, *ASM* appendicular skeletal muscle mass, *BIA* bioelectrical impedance analysis, *BMI* body mass index, *DXA* dual-energy X-ray absorptiometry, *Ht*^*2*^ height in metres squared, *PPA* positive predictive agreement (% of participants who were classified into the low muscle mass or sarcopenia criteria), *NPA* negative predictive agreement (% of participants who were classified into not having low muscle mass or sarcopenia criteria). Number of participants with BIA and DXA data (*n* = 3459)

## Discussion

The main finding from this study was that the overall prevalence of sarcopenia, defined as low muscle strength and mass, was very low (0.1–1.4%) and not different between middle-aged and older adults with or without NAFLD, regardless of the assessment (DXA versus BIA) or adjustment method for muscle mass (height^2^ versus BMI). Regarding the individual components of sarcopenia, the prevalence of low muscle strength was similar between NAFLD and non-NAFLD males and females (range 3.6–7.2%), but the prevalence of low muscle mass when adjusting for BMI versus height^2^ was different and in opposite directions, which was confirmed by the low agreement (Kappa 0.01–0.04) between the two adjustment methods (height^2^ versus BMI). Finally, for impaired physical function the risk (prevalence ratio) was 40 and 123% higher in males and females with NAFLD, respectively. Collectively, these findings suggest that in this select sample of UK middle-aged and older adults with NAFLD, muscle strength is not compromised compared to those without NAFLD, but they have low muscle mass when accounting for their increased adiposity, and impaired physical function.

The overall low prevalence of sarcopenia in our study in those with (and without) NAFLD is likely attributable to the demographic characteristics of the UK Biobank cohort. The mean age of participants in our study was 62.7 years (range 45–79) with 36% being under 60 years of age. Previous research in community dwelling middle-aged and older adults using the EWGSOP2 criteria to diagnose sarcopenia have reported the prevalence to range from 0.4 to 8.6% [27, 28] increasing to 8.1–20.0% in those > 70 years [29–31]. Despite evidence that the presence of chronic disease [32], including NAFLD [3], is associated with an increased risk of sarcopenia, we found no apparent difference between those with and without NAFLD, despite those with NAFLD having a higher prevalence of obesity and co-morbidities. The main reason for the lack of any differences in sarcopenia prevalence in our study is because muscle strength (the initial component of the sarcopenia definition) was not compromised in those with NAFLD. Previous studies examining the effects of NAFLD on muscle strength have reported mixed findings [16, 33] which may be related in part, to differences in the age of participants across studies and/or the potential confounding effects of body size (obesity). Indeed, there is some evidence that muscle (grip) strength is compromised in those with NAFLD when adjusted for BMI or weight [14, 33–35]. Although the current EWGSOP2 definition of sarcopenia does not recommend adjusting muscle strength for BMI or weight, when we adjusted absolute grip strength by BMI, we found it was 13.4% and 19.2% lower in males and females with NAFLD, respectively compared to those without NAFLD (data not shown). Based on these findings, further research is warranted to investigate whether normalising muscle strength for some measure of body size should be considered when evaluating the prevalence of low muscle strength, particularly in populations with increased adiposity, and what cut-offs should be used to define low muscle strength.

Another key finding was that the prevalence of low muscle mass in those with and without NAFLD was significantly different (and in the opposite direction) when adjusting for BMI versus height^2^, which is in concordance with the poor agreement (Kappa 0.01–0.04) we observed between the two adjustment methods. On average, those with NAFLD had significantly higher BMI and absolute (kg) muscle mass but when adjusted for BMI the prevalence ratio of low muscle mass was significantly higher in those with NAFLD; the opposite finding was observed when adjusted for height^2^. Previous studies have also reported a higher prevalence of low muscle mass when adjusting for BMI in those with NAFLD (8.7% vs 3.6%) [36] and no differences when adjusting for height^2^ [5]. This suggests that it is important to account for adiposity when evaluating the influence of NAFLD on muscle mass, especially in overweight and obese cohorts.

Another interesting observation from our study was that the overall prevalence of low muscle mass (and sarcopenia) was 1.1 to 6.5-fold higher when assessed by DXA compared to BIA, irrespective of NAFLD status or the method of adjustment. This is in alignment with the moderate agreement between DXA and BIA for quantifying low muscle mass in our cohort (Kappa 0.38–0.58, *P* < 0.001). These findings are likely explained by the fact that estimates of FFM and appendicular lean mass are typically overestimated by BIA, but this may also vary according to BMI [37, 38]. In our study, to estimate ASM from BIA we utilised a previously published equation developed from 4350 UK Biobank participants who had both a BIA and DXA scan, but the authors did not report on the level of agreement between DXA ALM and BIA estimated ASM in this study [23]. However, another study from the UK Biobank which validated BIA with DXA in 905 men and women reported that BIA overestimated ASM compared to DXA by 2.5% (women) and 1.9% (men) [39]. This confirms previous findings that BIA likely underestimates the prevalence of low muscle mass compared to DXA if using the same cut-off values [40, 41].

Poor physical function (a key component of sarcopenia) is associated with a range of adverse outcomes including falls, disability, and mortality [10]. Our findings indicated NAFLD was associated with a 40–123% increased prevalence ratio of impaired function (self-reported slow walking speed), after adjusting for several confounders including age, physical activity, smoking status, and the presence of chronic diseases, which was significantly higher in females only. However, when including BMI as a confounder the prevalence of impaired function was not significantly different between those with and without NAFLD. This suggests that adiposity was also likely a key factor contributing to impaired function in those with NAFLD, which is consistent with previous research reporting that obesity is associated with a higher risk of functional impairment [42]. To date, there are mixed findings from the few studies that have assessed whether NAFLD adversely influences physical performance, but most studies have reported no adverse effects on measures such as gait speed [5, 12]. Given the limited evidence available, further research is warranted to evaluate the impact of NAFLD on physical function.

A key strength of this study was the assessment of sarcopenia using all three components in a relatively large Western population of middle-aged and older adults with and without NAFLD. Other strengths include the assessment of body composition by BIA and DXA and the assessment of liver fat by MRI liver imaging, which is considered the gold standard to quantify NAFLD [43]. There are however several limitations. First, the UK Biobank cohort has been reported to represent ‘healthy volunteer’ selection bias [44]. Second, this study included a subset of participants from the larger UK Biobank sample (> 500,000) and those that had imaging data collected in 2014 (~ 10,000) which could limit generalisability, but the characteristics of the participants in our study versus the whole cohort was comparable (proportion female 54.9% vs 54.4%; weight 75.9 kg vs 78.1 kg, BMI 26.6 kg/m^2^ vs 27.4 kg/m^2^; proportion white 93.7% vs 94.6%; diabetes diagnosis 5.6% vs 5.3%). Third, the overall low prevalence of sarcopenia of participants in this study limits our ability to draw definitive conclusions regarding the influence of NALFD on the risk of sarcopenia. Fourth, DXA was only available in a subset (47%) of participants. Fifth, the estimate of ASM from BIA based on the equation developed by Dodds et al. [23] likely overestimates appendicular muscle mass, which may introduce a systematic error (bias) for this measure. Sixth, although we adjusted for several relevant covariates, there may be other potential confounders not considered that could influence the findings (e.g. diet, certain medications). Dietary data for the 2014 time point was not available and therefore could not be considered in the analysis. Finally, physical function was self-reported and did not include relevant objective measures such as the 4-m walking (gait) speed test.

In summary, this study indicates that adults aged 45–79 years with NAFLD do not have an increased likelihood sarcopenia, regardless of the method of body composition assessment (DXA versus BIA) or adjustment (height^2^ versus BMI). This was likely due to absolute hand grip (muscle) strength not being compromised in those with NAFLD. However, NAFLD was associated with an increased risk for low muscle mass when adjusting for adiposity (BMI), but not for height^2^, and impaired physical function. These findings support the use of adiposity-based adjustments when assessing the risk for low muscle mass in adults with NAFLD. In view of the close association between NAFLD and adiposity, and the recent proposed consensus definition and recommendations related to sarcopenic obesity, future work investigating the prevalence and impact of sarcopenic obesity in this cohort is warranted [45]. Additionally, further research assessing physical function in NAFLD is needed to determine whether it should be considered as part of routine screening or assessment for this population given that impairment is strongly linked to many adverse health outcomes.

### Supplementary Information

Below is the link to the electronic supplementary material.

Supplementary file1 (DOCX 22 KB)
