# Correction to: Prevalence of Sarcopenia and Its Defining Components in Non-alcoholic Fatty Liver Disease Varies According to the Method of Assessment and Adjustment: Findings from the UK Biobank

**DOI:** 10.1007/s00223-024-01235-y

**Published:** 2024-06-05

**Authors:** Christine L. Freer, Elena S. George, Sze-Yen Tan, Gavin Abbott, David Scott, Robin M. Daly

**Affiliations:** 1 Institute for Physical Activity and Nutrition, School of Exercise and Nutrition Sciences, Deakin University, Geelong, VIC Australia; 2 School of Clinical Sciences at Monash Health, Monash University, Clayton, Australia

**Correction to: Calcifed Tissue International (2024) 114:592–602** 10.1007/s00223-024-01212-5

In this article, P values in two occurrences were incorrectly processed by the production team while incorporating the corrections during the correction process.

That is, all P values originally had 3 decimal places. But it was cut off as “ < 0.0” and “0.14” mistakenly in Table 1 and 2.

1. Incorrect Values: “Age (years)” columns, P values as “ < 0.0 “
  Correction Values: “Age (years)” columns, P values “ < 0.001”.
2. Incorrect Values: “Low muscle strength” columns, P values as “0.14 “
  Correction Values: “Low muscle strength” columns, P values “0.149”.

The correct version of Table 1 and 2 are given below. The original article has been corrected.

**Table 1:** Characteristics of the study population according to NAFLD status and sex

	Females		*P* value	Males		*P* value
	Non-NAFLD	NAFLD		Non-NAFLD	NAFLD	
*n*	3294	697		2390	885	
Age (years)	61.9 ± 7.4	62.9 ± 7.0	< 0.001	63.7 ± 7.8	63.0 ± 7.7	0.020
Height (cm)	162.9 ± 6.2	161.9 ± 6.2	< 0.001	175.8 ± 6.6	175.8 ± 6.5	0.933
Weight, kg	67.2 ± 11.6	80.9 ± 14.7	< 0.001	80.2 ± 11.6	92.8 ± 14.8	< 0.001
BMI, kg/m^2^	25.3 ± 4.2	30.8 ± 5.1	< 0.001	25.9 ± 3.3	30.0 ± 4.2	< 0.001
Ethnicity^a^, *n* (%)						
White	3065 (93.2)	656 (94.4)	0.570	2234 (93.6)	832 (94.5)	0.295
Non-White	223 (6.8)	39 (5.6)		153 (6.4)	49 (5.5)	
Smoking status, *n*						
Never, *n* (%)	2310 (70.2)	461 (66.3)	0.126	1534 (64.3)	521 (59.1)	0.012
Previous, *n* (%)	885 (26.9)	211 (30.4)		771 (32.3)	317 (36.0)	
Current, *n* (%)	94 (2.9)	23 (3.3)		82 (3.4)	43 (4.9)	
Comorbidities^b^, *n* (%)						
1	826 (25.1)	263 (37.7)	< 0.001	772 (32.3)	340 (38.4)	< 0.001
≥ 2	99 (3.0)	81 (11.6)		156 (6.5)	125 (14.1)	
PA, MET- min/week^c^	2961 ± 3249	2375 ± 2477	< 0.001	3248 ± 3722	2330 ± 2966	< 0.001
BIA						
ASM, kg	17.5 ± 2.1	19.2 ± 2.7	< 0.001	25.0 ± 3.3	27.8 ± 4.2	< 0.001
ASM/Ht^2^	6.60 ± 0.70	7.33 ± 0.35	< 0.001	8.08 ± 0.88	8.98 ± 1.13	< 0.001
ASM/BMI	0.700 ± 0.080	0.629 ± 0.062	< 0.001	0.970 ± 0.098	0.931 ± 0.091	< 0.001
DXA^d^						
ALM, kg	17.2 ± 2.5	18.5 ± 3.0	< 0.001	25.0 ± 3.3	27.1 ± 4.1	< 0.001
ALM/Ht^2^	6.47 ± 0.79	7.10 ± 1.00	< 0.001	8.06 ± 0.87	8.72 ± 1.04	< 0.001
ALM/BMI	0.683 ± 0.096	0.608 ± 0.078	< 0.001	0.966 ± 0.117	0.908 ± 0.099	< 0.001
Grip strength, kg	24.6 ± 5.8	24.1 ± 6.1	0.062	40.1 ± 8.3	40.2 ± 8.2	0.685
Physical function, *n* (%)						
Slow	117 (3.6)	75 (10.8)	< 0.001	77 (3.2)	50 (5.6)	< 0.001
Steady	1596 (48.4)	461 (66.2)		1129 (47.2)	524 (59.2)	
Brisk	1580 (48.0)	160 (23.0)		1184 (49.5)	311 (35.1)	

Values represent mean ± SD or frequency counts and proportions (%) unless otherwise indicated

*ALM* appendicular lean mass, *ASM* appendicular skeletal muscle mass, *BIA* bioelectrical impedance analysis, *BMI* body mass index, *DXA* dual-energy X-ray absorptiometry, *H*^*2*^ height in metres squared, *MET* metabolic equivalents, *NAFLD* non-alcoholic fatty liver disease, *PA* physical activity, *PDFF* proton density fat fraction, *P* *P* value

^a^Number of participants with ethnicity data (*n* = 7251)

^b^Comorbidities included diagnosed diabetes, vascular/heart problems (heart attack, angina, stroke, high blood pressure) and cancer

^c^Number of participants with PA data (*n* = 6169)

^d^Number of participants with DXA data (*n* = 3459)

**Table 2:** Sex-specific prevalence of low muscle strength, low appendicular skeletal muscle mass according to BIA and DXA adjusting for height and BMI, and impaired physical function, in those with and without NAFLD and prevalence ratios (95% CI) according to NAFLD

	Non-NAFLD	NAFLD	Prevalence ratio (95% CI)					
			Model 1	*P* value	Model 2	*P* value	Model 3	*P* value
Females	*n* = 3294	*n* = 697						
Low muscle strength, *n* (%)	158 (4.8)	50 (7.2)	1.41 (0.96, 2.08)	0.080	1.33 (0.90, 1.96)	0.149	1.46 (0.96, 2.21)	0.079
Low muscle mass, *n* (%)								
* ASM/Ht*^*2 (BIA)*^	78 (2.4)	4 (0.6)	0.08 (0.01, 0.59)	0.013	0.08 (0.01, 0.57)	0.012	*–*	*–*
* ASM/BMI*^*(BIA)*^	12 (0.4)	15 (2.2)	5.10 (1.92, 13.54)	0.001	4.70 (1.79, 12.25)	0.002	*–*	*–*
Low muscle mass^a^, *n* (%)								
* ALM/Ht*^*2 (DXA)*^	126 (8.0)	8 (2.3)	0.19 (0.07, 0.51)	0.001	0.18 (0.07, 0.49)	0.001	*–*	*–*
* ALM/BMI*^*(DXA)*^	41 (2.6)	26 (7.6)	2.55 (1.41, 4.61)	0.002	2.21 (1.22, 4.02)	0.009	*–*	*–*
Impaired function, *n* (%)	117 (3.6)	75 (10.8)	2.65 (1.84, 3.80)	< 0.001	2.23 (1.56, 3.20)	< 0.001	1.02 (0.68, 1.52)	0.923
Males	*n* = 2390	*n* = 885						
Low muscle strength, *n* (%)	109 (4.6)	32 (3.6)	0.73 (0.48, 1.13)	0.161	0.77 (0.50, 1.19)	0.238	0.83 (0.52, 1.32)	0.422
Low muscle mass, *n* (%)								
* ASM/Ht*^*2 (BIA)*^	227 (9.5)	11 (1.2)	0.11 (0.05, 0.22)	< 0.001	0.11 (0.06, 0.23)	< 0.001	*–*	*–*
* ASM/BMI*^*(BIA)*^	59 (2.5)	39 (4.4)	1.65 (1.07, 2.53)	0.023	1.74 (1.12, 2.70)	0.013	*–*	
Low muscle mass^a^, *n* (%)								
* ALM/Ht*^*2 (DXA)*^	112 (10.0)	13 (3.1)	0.29 (0.16, 0.53)	< 0.001	0.29 (0.16, 0.54)	< 0.001	*–*	*–*
* ALM/BMI*^*(DXA)*^	67 (6.0)	50 (12.0)	1.60 (1.08, 2.37)	0.018	1.59 (1.08, 2.35)	0.019	*–*	*–*
Impaired function, *n* (%)	77 (3.2)	50 (5.6)	1.68 (1.14, 2.47)	0.008	1.40 (0.95, 2.07)	0.086	0.77 (0.52, 1.15)	0.205

Values are presented as number and proportions (%) or the prevalence ratio with the 95% confidence intervals (CI)

*ALM* appendicular lean mass, *ASM* appendicular skeletal muscle mass, *BIA* bioelectrical impedance analysis, *BMI* body mass index, *DXA* dual-energy X-ray absorptiometry, *Ht*^*2*^ height in metres squared

^a^Number of participants with DXA data: females *n* = 1579 non-NAFLD and *n* = 344 NAFLD; males *n* = 1119 non-NAFLD and *n* = 417 NAFLD

Model 1: unadjusted. Model 2: adjusted for age, physical activity, presence of comorbidities and smoking status. Model 3: adjusted for age, physical activity, presence of comorbidities, smoking status and BMI

