# Supplementary material for: Prevalence of Sarcopenia and Its Defining Components in Non-alcoholic Fatty Liver Disease Varies According to the Method of Assessment and Adjustment: Findings from the UK Biobank
Source: Calcif Tissue Int. 2024 Apr 28;114(6):592–602. doi: 10.1007/s00223-024-01212-5 (PMC11090922; doi:10.1007/s00223-024-01212-5)
Supplement: Supplementary file 1 — Supplementary file1 (DOCX 22 KB) [file 223_2024_1212_MOESM1_ESM.docx]

**Supplementary Table 1:** Characteristics of the participants according to body composition assessment method and sex.

|  | **BIA** | | **DXA** | |
| --- | --- | --- | --- | --- |
|  | **Females** | **Males** | **Females** | **Males** |
| n | 3991 | 3275 | 1923 | 1536 |
| Age (years) | 62.1 ± 7.3 | 63.5 ± 7.8 | 61.8 ± 7.4 | 63.1 ± 7.7 |
| Height (cm) | 162.8 ± 6.2 | 175.8 ± 6.6 | 162.6.8 ± 6.3 | 175.9 ± 6.6 |
| Weight, kg | 69.6 ± 13.2 | 83.6 ± 13.7 | 69.8 ± 13.2 | 83.8 ± 13.7 |
| BMI, kg/m^2^ | 26.3 ± 4.8 | 27.0 ± 4.0 | 26.4 ± 4.8 | 27.1 ± 3.9 |
| Ethnicity^a^, n (%) |  |  |  |  |
| White | 3721 (93.4) | 3066 (93.8) | 1780 (92.8) | 1423 (92.8) |
| Non-White | 262 (6.6) | 202 (6.2) | 138 (7.2) | 110 (7.2) |
| Smoking status, n |  |  |  |  |
| Never, n (%) | 2771 (69.6) | 2055 (62.9) | 1316 (68.6) | 959 (62.6) |
| Previous, n (%) | 1096 (27.5) | 1088 (33.3) | 541 (28.2) | 509 (33.2) |
| Current, n (%) | 117 (2.9) | 125 (3.8) | 61 (3.2) | 64 (4.2) |
| Comorbidities, n (%) |  |  |  |  |
| 1 comorbidity | 1089 (27.3) | 1112 (34.0) | 528 (27.5) | 532 (34.6) |
| ≥2 comorbidities | 180 (4.5) | 281 (8.6) | 105 (5.5) | 137 (8.9) |
| PA, MET- min/week^b^ | 2865 ± 3143 | 3001± 3557 | 2839 ± 3148 | 2963 ± 3601 |

BIA, bioelectrical impedance analysis; BMI, body mass index; DXA, dual-energy X-ray absorptiometry; H^2^, height in metres squared; PA, physical activity; Values represent mean ± SD or frequency counts and proportions (%) unless otherwise indicated. ^a^ Number of participants with ethnicity data: BIA, n= 7251; DXA, n=3451. ^b^ Number of participants with physical activity data: BIA, n=6169; DXA, n=2947.

**Supplementary Table 2:** Sex-specific prevalence of low muscle strength, low appendicular skeletal muscle/lean mass (ASM and ALM according to BIA and DXA) adjusting for height and BMI, and impaired physical function, in those with and without NAFLD and the adjusted prevalence ratios (95% CI) according to NAFLD in those participants based on both DXA and BIA.

|  |  |  | **Prevalence Ratio (95% CI)** | | | | | |
| --- | --- | --- | --- | --- | --- | --- | --- | --- |
|  | **Non-NAFLD** | **NAFLD** | **Model 1** | *P* | **Model 2** | *P* | **Model 3** | *P* |
| **Females** | n=1579 | n=344 |  |  |  |  |  |  |
| Low muscle strength, n (%) | 100 (6.3) | 37 (10.8) | 1.68 (1.09, 2.60) | 0.019 | 1.50 (0.97, 2.34) | 0.071 | 1.70 (1.04, 2.80) | 0.035 |
| Low muscle mass, n (%) |  |  |  |  |  |  |  |  |
| *ASM/Ht^2 (BIA)^* | 28 (1.8) | 3 (0.9) | 0.24 (0.32, 1.77) | 0.162 | 0.22 (0.03, 1.65) | 0.142 | - | - |
| *ASM/BMI ^(BIA)^* | 4 (0.3) | 8 (2.3) | 11.9 (2.32, 61.3) | 0.003 | 9.98 (1.98, 50.29) | 0.005 | - | - |
| Low muscle mass^a^, n (%) |  |  |  |  |  |  |  |  |
| *ALM/Ht^2 (DXA)^* | 126 (8.0) | 8 (2.3) | 0.19 (0.07, 0.51) | 0.001 | 0.18 (0.07, 0.49) | 0.001 | - | - |
| *ALM/BMI ^(DXA)^* | 41 (2.6) | 26 (7.6) | 2.55 (1.40, 4.61) | 0.002 | 2.21 (1.22, 4.02) | 0.009 | - | - |
| Impaired function, n (%) | 54 (3.4) | 38 (11.1) | 2.97 (1.80, 4.92) | <0.001 | 2.45 (1.48, 4.08) | 0.001 | 1.11 (0.64, 1.94) | 0.698 |
| **Males** | n=1119 | n=417 |  |  |  |  |  |  |
| Low muscle strength, n (%) | 68 (6.1) | 21 (5.0) | 0.71 (0.40, 1.23) | 0.222 | 0.77 (0.44, 1.34) | 0.351 | 0.85 (0.47, 1.53) | 0.584 |
| Low muscle mass, n (%) |  |  |  |  |  |  |  |  |
| *ASM/Ht^2 (BIA)^* | 98 (8.8) | 5 (1.2) | 0.09 (0.03, 0.29) | <0.001 | 0.10 (0.03, 0.31) | <0.001 | - | - |
| *ASM/BMI ^(BIA)^* | 34 (3.0) | 12 (2.9) | 0.85 (0.42, 1.72) | 0.653 | 0.94 (0.46, 1.93) | 0.866 | - | - |
| Low muscle mass^a^, n (%) |  |  |  |  |  |  |  |  |
| *ALM/Ht^2 (DXA)^* | 112 (10.0) | 13 (3.1) | 0.29 (0.16, 0.53) | <0.001 | 0.29 (0.16, 0.54) | <0.001 | - | - |
| *ALM/BMI ^(DXA)^* | 67 (6.0) | 50 (12.0) | 1.60 (1.08, 2.37) | 0.018 | 1.59 (1.08, 2.35) | 0.019 | - | - |
| Impaired function, n (%) | 39 (3.5) | 22 (5.3) | 1.50 (0.83, 2.68) | 0.176 | 1.25 (0.69, 2.27) | 0.464 | 0.70 (0.38, 1.27) | 0.241 |

Values are presented as number and proportions (%) or the prevalence ratio with the 95% confidence intervals (CI). ALM, appendicular lean mass; ASM, appendicular skeletal muscle mass; BIA, bioelectrical impedance analysis; BMI, body mass index; DXA, dual-energy X-ray absorptiometry; Ht^2^, height in metres squared.

Model 1: unadjusted.

Model 2: adjusted for age, physical activity, presence of comorbidities and smoking status.

Model 3: adjusted for age, physical activity, presence of comorbidities, smoking status and BMI.
